# Prescription Trends in Complex Regional Pain Syndrome: A Retrospective Case–Control Study

**DOI:** 10.3390/brainsci13071012

**Published:** 2023-06-30

**Authors:** Suzanna Shermon, Kimberly M. Fazio, Richard Shim, Alaa Abd-Elsayed, Chong H. Kim

**Affiliations:** 1 MetroHealth Medical Center, Case Western Reserve University, 4229 Pearl Road, Cleveland, OH 44109, USA; kfazio@metrohealth.org (K.M.F.); ckim3@metrohealth.org (C.H.K.); 2 School of Medicine, Case Western Reserve University, Cleveland, OH 44109, USA; wxs404@case.edu; 3 Department of Anesthesiology, University of Wisconsin, Madison, WI 53706, USA; alaaawny@hotmail.com

**Keywords:** CRPS, opioids, benzodiazepines, NSAIDs, neuropathic-pain medicine

## Abstract

Objective: The objective of this study was to evaluate discrepancies in prescription trends for analgesic medications in complex regional pain syndrome (CRPS) patients based on recommendations in the literature. Design: We conducted a retrospective case–control study. Subjects: A total of 2510 CRPS patients and 2510 demographic-matched controls participated in this study. Methods: The SlicerDicer feature in Epic was used to find patients diagnosed with CRPS I or II between January 2010 and November 2022. An equal number of age-, gender-, and race-matched controls without a CRPS diagnosis were retracted from Epic. General and CRPS-associated prescription frequencies for the following classes were retrieved for both cases and controls: benzodiazepines, bisphosphonates, calcitonin, capsaicin, neuropathic pain medications, NSAIDs, opioids, and steroids. Results: A total of 740 (29%) CRPS patients and 425 (17%) controls were prescribed benzodiazepines (95% CI 0.1–0.15), 154 (6.1%) CRPS patients and 52 (2.1%) controls were prescribed capsaicin (95% CI 0.03–0.05), 1837 (73%) CRPS patients and 927 (37%) controls were prescribed neuropathic pain medications (95% CI 0.05–0.34), 1769 (70%) CRPS patients and 1217 (48%) controls were prescribed opioids (95% CI 0.19–0.25), 1095 (44%) CRPS patients and 1217 (48%) controls were prescribed steroids (95% CI 0.08–0.14), and 1638 (65%) CRPS patients and 1765 (70%) controls were prescribed NSAIDs (95% CI −0.08–0.02), *p* < 0.001 for all classes. With CRPS-associated prescriptions, (95% CI 0.05–0.16, *p* < 0.001) more CRPS patients were prescribed opioids (N = 398, 59%) than controls (N = 327, 49%). Conclusions: CRPS is difficult to treat with significant variance in suggested treatment modalities. Based on the results of our study, there is a divergence between some published recommendations and actual practice.

## 1. Introduction

Complex regional pain syndrome (CRPS) is a rare and painful neurologic condition often resulting from trauma with two subtypes, CRPS I (previously called reflex sympathetic dystrophy) and CRPS II (previously known as causalgia), which differ by the lack of or presence of a major nerve injury, respectively [1]. CRPS is a diagnosis of exclusion based on history, physical exam, and Budapest Criteria [2,3]. Symptoms can include allodynia, hyperesthesia, skin color change, temperature, sweating asymmetry, motor and trophic changes, decreased range of motion, and edema [2,3].

G-protein coupled receptors (GPCRs) may play a significant role in the development of neurogenic inflammation, sensitization of nociceptors, and modulation of pain signaling pathways [4,5], which can impact the development and treatment of CRPS. Recent research focuses on the effects of GPCRs, specifically cannabinoid and adenosine receptors, on the ion channel transient receptor potential vanilloid 1 (TRPV1), which is involved in both thermal and inflammatory pain regulation. These studies conducted in rat models have shown promising results of modulation of the pain pathway via GPCR modulation of TRPV1 [6,7]. However, despite these important advancements, the complex pathophysiology of CRPS still leaves treatment options elusive without specific guidelines for the management of this condition.

In a clinical setting, certain medications have been shown to be effective. Neuropathic pain medications, primarily gabapentin and amitriptyline, have some evidence for efficacy in pain reduction [1,8,9,10,11,12,13,14,15]. Non-steroidal anti-inflammatory drugs (NSAIDs) and bisphosphonates may be effective in the acute phases of CRPS [1,8,9,10,11,12,16,17,18,19,20,21,22,23]. Lower-quality supporting evidence exists for the use of calcitonin and glucocorticoids in this syndrome [1,8,9,10,11,12,19,20,21]. Meanwhile, there is no supporting evidence for the use of opioids or benzodiazepines [8,12,24].

The objective of this study was to evaluate the prescription trends for analgesic medications in CRPS patients and compare them to non-CRPS controls at our institution. The selected drug classes for this study have either shown symptom relief for CRPS patients [2,7,8,9,10,11,12,13,14,15,16,17,18,19,20,21,25,26] or are frequently used in chronic or neuropathic pain conditions [11,12,13]. We hypothesize that prescription trends in CRPS patients will be discrepant from recommendations in the literature.

## 2. Methods

This study is a retrospective case–control performed using the electronic medical record (EMR), specifically the SlicerDicer feature in the Epic, to find patients diagnosed with CRPS I or II between January 2010 and November 2022 at an urban academic teaching hospital. SlicerDicer is a cohort-query tool that allows users to look at general trends in various categories related to patient care at any institution using Epic as its EMR. The following search criteria were used to locate patients via diagnosis or medical history: ‘complex regional pain syndrome I, unspecified,’ ‘complex regional pain syndrome of upper limb,’ complex regional pain syndrome, type II,’ ‘complex regional pain syndrome of lower limb,’ and ‘complex regional pain syndrome of hand.’ These were all the possible different CRPS diagnoses found in our institution’s EMR. The search yielded 2510 CRPS patient cases within this time frame.

In collaboration with medical informatics, the medical record numbers (MRNs) and demographic data were retrieved for the 2510 CRPS cases. An equal number (2510) of age-, gender-, and race-matched controls for each individual subject in the case category without a CRPS diagnosis were supplied.

Medication data for prescriptions between January 2010 and November 2022 were evaluated for the 5020 cases and controls. The medication classes chosen were based on published recommendations for CRPS patients as well as some medications that may be prescribed for those with chronic pain [1,8,9,10,11,12,13,14,15,16,17,18,19,20,21,22,23,24]. Prescription frequencies for the following classes were retrieved for both cases and controls: benzodiazepines, bisphosphonates, calcitonin, capsaicin, neuropathic pain medications, NSAIDs, opioids, and steroids. To avoid confounders, filters isolated medications prescribed in an outpatient setting. Exclusions included medications prescribed during inpatient hospital stays and emergency department visits on day of and two weeks after any surgery and for cancer pain to further ensure that all examined medications were prescribed on an outpatient basis for non-cancer pain.

Specific medications belonging to each medication class were retrieved. For each subject, the first and last prescribed dates and dosages of each medication were obtained to assess the changes in dosage over time. The years that each medication was prescribed were also retrieved in order to assess longevity of use.

The first goal was to evaluate for a difference in prescription rates via drug class and specific medications between cases and matched controls. In addition to general prescription rates, a filter was applied to search for medications with a CRPS-associated diagnosis in Epic. Those cases were then compared to their corresponding controls. The second goal was to compare the longevity of medication use between the two groups. The third goal was to evaluate for change in dosage between the first and last prescription of each opioid in the CRPS group. The fourth goal was to examine prescription rates of opioids before and after 1 January 2017 to account for the release of the 2016 CDC guidelines for prescribing opioids for chronic pain [27].

For statistical analysis, a two-sample test for equality of proportions was run with a 95% confidence interval and a false discovery rate for multiple testing in order to determine a statistically significant difference between medication prescriptions in cases and controls and differences in prescriptions from prior to and after 2017. A Welch two-sample t-test with a 95% confidence interval and false discovery rate correction for multiple testing was performed to evaluate average longevity of use and statistical differences between cases and controls. A Wilcoxon rank sum test and a Pearson’s chi-squared test were run to evaluate demographic data. Significant results were considered at *p* < 0.05.

## 3. Results

This study consisted of 2510 CRPS cases and 2510 age-, gender-, and race-matched controls. The median age of subjects in both the case and control groups was 58, with a range from 47 to 66. Subjects were further subdivided into age categories, with the majority of cases and controls (N = 1408 per group (56%)) between 41 and 64 years old. A total of 725 (29%) in both groups were over 65 years old, 365 (15%) in both groups were between 19 and 40 years old, and 12 (0.5%) in both groups were under 18 years old. In each group, there were 1576 females (63%) and 934 males (37%). Both the cases and controls consisted of the following races in each group: Caucasian 1538 (61%), African American 701 (28%), ‘other’ 35 (1.4%), and ‘unknown’ 236 (9.4%). ‘American Indian/Alaska native,’ ‘Asian,’ ‘multiracial’ and ‘Native Hawaiian/Pacific Islander’ were grouped together into the ‘other’ category due to the small percentage of each of these races in this study. Anyone who declined to answer or put unknown as their race was grouped into the ‘unknown’ category. These results are in Table 1.

Comparisons between the number of subjects prescribed for each type of drug class can be found in Table 2a. There was a higher prescription rate (*p* < 0.001 for all) of the following drug classes for CRPS patients: benzodiazepines, capsaicin, neuropathic pain medications, opioids, and steroids. More specifically, in the 12-year study period, 740 (29%) CRPS patients and 425 (17%) controls were prescribed benzodiazepines (95% CI 0.1–0.15), 154 (6.1%) CRPS patients and 52 (2.1%) controls were prescribed capsaicin (95% CI 0.03–0.05), 1837 (73%) CRPS patients and 927 (37%) controls were prescribed neuropathic pain medications (95% CI 0.05–0.34), 1769 (70%) CRPS patients and 1217 (48%) controls were prescribed opioids (95% CI 0.19–0.25), and 1095 (44%) CRPS patients and 1217 (48%) controls were prescribed steroids (95% CI 0.08–0.14). There was a statistically significant difference (*p* < 0.001) in NSAID prescriptions; 1638 (65%) CRPS patients and 1765 (70%) controls were prescribed NSAIDs (95% CI −0.08–0.02). No significant differences were found in bisphosphonate and calcitonin prescriptions between the two groups. Additionally, 2211 (88%) CRPS patients and 2510 (100%) controls were prescribed at least one of these drug classes (*p* < 0.001).

A filter was applied to search for medications specifically associated with a CRPS diagnosis in Epic. A total of 670 of the cases had associated CRPS medications. They were compared with 670 controls. As shown in Table 2b, more (95% CI 0.05–0.16, *p* < 0.001) CRPS patients were prescribed opioids (N = 398, 59%) than were controls (N = 327, 49%). More (95% CI 0.26–0.37, *p* < 0.001) CRPS patients (N = 460, 69%) were also prescribed neuropathic pain medications than the control group (N = 250, 37%). Bisphosphonates (95% CI −0.05–0.02, *p* < 0.001), NSAIDs (95% CI −0.58–0.48, *p* < 0.001), steroids (95% CI −0.27–0.18, *p* < 0.001), and capsaicin (95% CI −0.03–0, *p* < 0.05) were all prescribed at significantly lower rates in the CRPS group than in the control group. No significant differences were noted in benzodiazepine or calcitonin prescriptions between the two groups.

Differences in the longevity of use of each drug class between cases and controls were assessed. Table 3 shows the average number of years and standard deviation (SD) that patients were taking each medication class. The medication classes prescribed for over a year in the CRPS group were neuropathic pain medication (4.06 (5.18)), NSAIDs (3.11 (4.25)), opioids (3.95 (5.21)), and steroids (1.07 (1.95)). In the control group, neuropathic pain medications (1.35 (3.17)), NSAIDs (2.21 (3.08)), and opioids (1.3 (2.57)) were prescribed for longer than a year. Longer prescription durations in the CRPS group compared to the control group were seen in the following: benzodiazepines (95% CI 0.3–0.48, *p* < 0.001), bisphosphonates (95% CI 0–0.07, *p* < 0.05), capsaicin (95% CI 0.04–0.07, *p* < 0.001), neuropathic pain medications (95% CI 2.5–3, *p* < 0.001), NSAIDs (95% CI 0.7–1.1, *p* < 0.001), opioids (95% CI 2.4–2.9, *p* < 0.001), and steroids (95% CI 0.32–0.51, *p* < 0.001).

For the CRPS group only, the first and last doses of prescribed opioids were compared to assess for changes in dosage over time (Table 4). A total of 3930 prescriptions were evaluated at each time point. There were no significant changes in doses over time for any of the opioids. Hydrocodone appears to be significant based on its *p* value, but, when evaluating the false discovery rate correction for multiple testing, there is no significant difference between the first and last prescribed doses.

For both cases and controls, we examined differences in prescription rates of opioids from before and after 1 January 2017 to assess the impact of the 2016 CDC guidelines for prescribing opioids for chronic pain [27]. Opioid prescriptions decreased from 1470 (79%) cases before 2017 to 676 (50%) after (95% CI −0.32–−0.26, *p* < 0.001). In the control group, opioid prescriptions decreased from 905 (51%) before 2017 to 427 (30%) after 2017 (95% CI −0.25–−0.18, *p* < 0.001). The results are listed in Table 5.

## 4. Discussion

There are 2510 CRPS cases and 2510 controls with no significant difference in demographics. Each control was matched in age, gender, and race. As shown in Table 1, the average age of our subjects was 58 years old, with the majority between 41 and 64 years old (56%). A higher prevalence of female gender (63%) and Caucasian race (61%) was found in this study. These data are consistent with several epidemiological studies showing potential associations between the Caucasian race and female gender in CRPS [2,28,29]. Some discrepancies in the age of onset of CRPS exist in the literature. Two studies suggest that CRPS peaks from ages 45 to 55, which is consistent with the results of our study [28,29]. A different epidemiological study suggests that the average age of onset in CRPS is anywhere from 70 to 79 years old [30], which may also be congruent with our results that show the second most common CRPS age group is those over 65 years old (29%).

Our results show that there is a high prevalence of opioid prescriptions in the CRPS population, which is surprising since opioids are not generally recommended for this syndrome [11,31]. Only one RCT has been conducted studying controlled-release morphine on pain reduction in CRPS and found that there was no difference between the opioid and placebo groups [24]. Given how difficult this condition is to treat, there is a growing consensus that opioids may be a reasonable second or third option at low doses for a short period of time [8]. Contradictory to this information, the majority of CRPS patients were on opioids for multiple years in our study. Table 2a shows that 70% of CRPS patients have been prescribed opioids, which is significantly more than prescriptions for the 48% of controls. Table 2b shows that 59% of CRPS cases have a CRPS-associated opioid prescription, which is also significantly more than 49% of controls. These results are similar to a different study that found opioid use in 64% of CRPS subjects [32]. Additionally, CRPS patients are also prescribed opioids for a significantly longer duration (3.95 years on average) than controls (1.3 years on average), as seen in Table 3. These results are concerning, as long-term opioid use can worsen allodynia and hyperpathia [8,33]. These results may be attributed to opioids being prescribed for acute pain but then continued for any refractory, chronic pain [33]. Table 4 shows no significant dose change amongst any of the examined opioids from the first to last prescribed doses. There is some evidence that neuropathic pain does not respond as well to opioids as does nociceptive pain, which is why dose escalation of this drug class would not be useful [8,34,35]. The results of our study are consistent with these suggestions since there was no significant escalation of opioids over time. However, there were also no significant dose decreases despite suggestions for tapering due to the addictive properties of these medications [33].

Given the high rates of opioid prescriptions in both the case and control groups, we explored the role of the 2016 CDC guidelines on opioid prescription rates [27]. Total prescriptions for each drug class were compared prior to and after 2017, independently for cases and controls (Table 5). As suspected, opioid prescriptions decreased significantly after the release of the guidelines in both cases and controls. It is also worth noting that prescription rates for opioids were higher at all comparative time points in the CRPS group compared to the controls and that even after the release of the guidelines and other evidence-based recommendations against the use of opioids in this syndrome, half of the CRPS patients were still prescribed opioids after 2017 [8,24,27,32,33]. Since both the cases and controls showed a significant decrease in opioids from prior to 2017 to after, we cautiously attribute these results to the 2016 CDC guidelines [27].

Benzodiazepines are another drug class commonly prescribed for chronic pain despite no supporting evidence [36]. In patients with chronic, non-cancer pain, benzodiazepine use was associated with greater pain severity [36]. Another study found that patients taking benzodiazepines reported greater perceived pain severity and depressive symptoms compared to patients not taking them [37]. Our results in Table 2a suggest that benzodiazepines are prescribed significantly more often to CRPS patients (29%) than controls (17%) on a general basis. In Table 2b, 15% of cases had a CRPS-associated diagnosis to their benzodiazepine prescription, which was not significantly different from the control group, but still more than anticipated. Table 3 shows that benzodiazepines are prescribed for significantly longer periods in CRPS cases (0.76 years) than controls (0.37 years). Since there are no recommendations for benzodiazepine use in CRPS, the longevity and high frequency of prescriptions are possibly associated with the psychiatric symptoms related to the chronic pain experienced in CRPS and a developed dependency on these medications [33,34,35].

NSAIDs are often used for prophylaxis and rescue in some CRPS patients despite minimal evidence [8]. One RCT showed no significant difference in pain scores or edema after treatment with IV parecoxib vs. placebo in CRPS [38]. Another study compared the use of prednisolone with piroxicam for one month and found only mild improvements in CRPS symptoms noted in the NSAID group [17]. Table 2a,b and Table 3 indicate that CRPS patients are prescribed fewer NSAIDs than controls but for a longer duration of time. These results may possibly be due to NSAIDs providing adequate pain relief to this percentage of cases, especially given that they were prescribed this drug class for multiple years, which is consistent with the views of some experts that NSAIDs are effective in CRPS [8].

Neuropathic pain medications consist mostly of anti-depressants and anticonvulsants with limited evidence for use in CRPS [13]. Our study indicates that neuropathic pain medications were prescribed significantly more to CRPS patients than to controls (Table 2a,b). One randomized controlled study (RCT) showed that gabapentin significantly reduced pain and sensory deficit in those with CRPS [14]. A different RCT demonstrated improvement in pain scores and quality of life in various neuropathic conditions, including CRPS, after gabapentin administration [13]. An RCT comparing gabapentin vs. amitriptyline suggested that there is no significant difference between the two medications in providing analgesia in CRPS I [15]. CRPS patients are on neuropathic pain medications for an average of 4.06 years, which is significantly longer than the controls, who are on neuropathic pain medications for an average of 1.35 years (Table 3). Our results are reflective of some of the literature and indicate that neuropathic pain medications are a common adjuvant in CRPS, which may potentially show their effectiveness despite limited evidence [1,12,13,14,15,39].

Interestingly, very few CRPS patients were prescribed bisphosphonates or calcitonin despite recommendations in the literature [12,13,14,15,16,17,18,19,20,21,22,23,40]. Prescription rates of bisphosphonates and calcitonin in CRPS did not differ significantly from prescription rates in the general population (Table 2a), and CRPS-associated bisphosphonates and calcitonin were each prescribed to only one CRPS patient (Table 2b). Alendronate was the most commonly prescribed bisphosphonate (Supplemental Data). Two RCTs showed that alendronate helped with improving pain scores, swelling, and range of motion in CRPS patients [38,41]. Bisphosphonates may be effective in the acute phases of CRPS, especially with abnormal uptake on bone scans [13,24]. Meanwhile, the evidence for calcitonin utility in CRPS is mixed [42,43]. While no formal recommendations exist for the dose and duration of bisphosphonate use in CRPS, one meta-analysis showed that most studies focusing on this topic administered bisphosphonates anywhere from 3 days to 8 weeks [22]. Average bisphosphonate use in our study for the cases falls within that time frame and may be shorter for age-matched controls since they are younger than the typical osteoporosis population (Table 3). Possible explanations for low prescription rates of these medications may include them not typically being used for analgesia and having an unclear mechanism for pain relief in CRPS [1,10,11,40,44].

Capsaicin is helpful in some conditions resulting in neuropathic pain but has been less studied in CRPS, possibly due to the severe burning symptoms that result from this topical medication [8,45,46]. One study showed an improvement in CRPS pain scores after the application of high-dose capsaicin. However, in order for patients to be able to tolerate capsaicin, they received regional anesthesia prior to its application [47]. A case report suggested that capsaicin actually worsens the symptoms of CRPS due to its nociceptive stimulation [48]. Our results indicate that general and CRPS-associated prescription rates in the CRPS group were significantly higher and longer (Table 2a,b and Table 3) than capsaicin prescriptions in the general population, despite controversy about the use of this medication in CRPS [8,47,48]. However, our results also show that prescription rates remain low and short for both cases and controls, possibly due to the intolerable, painful side effects of this medication.

Supporting evidence for the use of glucocorticoids in CRPS is weak and long-term toxicities outweigh potential benefits [49]. There are several small randomized trials showing the potential benefit of the use of steroids in early CRPS [17,19,50]. Table 2a shows that CRPS patients are prescribed steroids significantly more than non-CRPS controls. Table 2b, however, shows that CRPS-associated steroid prescriptions are significantly lower in the CRPS group than in the control group. Steroids are prescribed for significantly longer in the CRPS group than in the control group, as shown in Table 3. The long duration of use and discrepancy with CRPS-associated prescriptions suggests that patients in our study were possibly prescribed steroids for conditions other than CRPS on a general basis, especially since short-term use has been proposed in the literature [17,19,50]. The 10% prescription rate for CRPS-associated medications suggests that steroids are not often used to treat this syndrome.

There are several limitations to this study. It was difficult to ensure that CRPS was properly diagnosed with Budapest criteria [3] because the study was retrospective. Additionally, it is unclear how helpful these medication classes actually were to the case group for CRPS symptomatology. Potential conclusions on their usefulness were, therefore, drawn from prescription rates and longevity of use. It was also difficult to ascertain the true percentage of each medication that was specifically prescribed for CRPS. Additionally, the results in Table 2b are dependent on providers choosing CRPS as the diagnosis to associate with these conditions rather than any other pain-related diagnosis that these symptoms may have been classified as. Another limitation is not knowing which specific conditions these medications were prescribed for in both the cases and controls. Future studies could conduct a similar project prospectively by following CRPS patients over time in order to determine what treatment modalities are being used and compare that to the general population. This would eliminate several limitations of our study by ensuring that CRPS patients are properly diagnosed via the Budapest criteria and confirming that medications are prescribed for CRPS and not any other condition. Additionally, other treatment modalities can be examined, such as therapies or more invasive procedures, such as sympathetic blocks or dorsal root ganglion stimulations. If performed prospectively, future studies can also evaluate how helpful these treatment modalities are to CRPS patients.

## 5. Conclusions

CRPS is difficult to treat with significant variance in the suggested treatment modalities. Based on the results of our study, there is a deviation from recommendations in the existing literature to the medication prescriptions seen in actual practice. These discrepancies are likely multifactorial. Our study highlights the clinical lack of consensus and awareness on current practice evidence for this condition, while other ongoing research continues to address our incomplete understanding of the pain and inflammatory pathways associated with CRPS.

## Figures and Tables

**Table 1:** Demographic information for CRPS cases and age-, sex-, and race-matched control group.

Characteristic	CRPS, N = 2510 ^1^	Control, N = 2510 ^1^	*p*-Value ^2^
Age	58 (47, 66)	58 (47, 66)	>0.9
age_category			>0.9
0–18	12 (0.5%)	12 (0.5%)	
19–40	365 (15%)	365 (15%)	
41–64	1408 (56%)	1408 (56%)	
65+	725 (29%)	725 (29%)	
Sex			>0.9
F	1576 (63%)	1576 (63%)	
M	934 (37%)	934 (37%)	
Race			>0.9
Unknown	236 (9.4%)	236 (9.4%)	
Other	35 (1.4%)	35 (1.4%)	
African American	701 (28%)	701 (28%)	
Caucasian	1538 (61%)	1538 (61%)	

^1^ Median (IQR); n (%); ^2^ Wilcoxon rank sum test; Pearson’s Chi-squared test.

**Table 2:** (a) Differences between general prescription rates of various medication classes amongst CRPS cases and controls. (b) CRPS-associated prescriptions in various medication classes amongst CRPS cases compared with matched controls.

2a	CRPS, N = 2510 ^1^	Control, N = 2510 ^1^	Difference ^2^	95% CI ^2,3^	*p*-Value ^2^	q-Value ^4^
Medication Class						
None	299 (12%)	0 (0%)	0.1191235060	0.11, 0.13	<0.001	<0.001
Benzodiazepines	740 (29%)	425 (17%)	0.1254980080	0.10, 0.15	<0.001	<0.001
Bisphosphonates	108 (4.3%)	92 (3.7%)	0.0063745020	0.00, 0.02	0.3	0.3
Calcitonin	5 (0.2%)	3 (0.1%)	0.0007968127	0.00, 0.00	0.7	0.7
Capsaicin	154 (6.1%)	52 (2.1%)	0.0406374502	0.03, 0.05	<0.001	<0.001
Neuropathic pain medications	1837 (73%)	927 (37%)	0.3625498008	0.34, 0.39	<0.001	<0.001
Nonsteroidal Anti-inflammatory Agents (NSAIDs)	1638 (65%)	1765 (70%)	−0.0505976096	−0.08, −0.02	<0.001	<0.001
Opioids	1769 (70%)	1217 (48%)	0.2199203187	0.19, 0.25	<0.001	<0.001
Steroids	1095 (44%)	817 (33%)	0.1107569721	0.08, 0.14	<0.001	<0.001
2b	CRPS, N = 670 ^1^	Other, N = 670 ^1^	Difference ^2^	95% CI ^2,3^	*p*-value ^2^	q-value ^4^
Medication Class						
Benzodiazepines	101 (15%)	110 (16%)	−0.013432836	−0.05, 0.03	0.5	0.6
Bisphosphonates	1 (0.1%)	24 (3.6%)	−0.034328358	−0.05, −0.02	<0.001	<0.001
Calcitonin	1 (0.1%)	0 (0%)	0.001492537	0.00, 0.01	>0.9	>0.9
Capsaicin	7 (1.0%)	19 (2.8%)	−0.017910448	−0.03, 0.00	0.029	0.039
Neuropathic pain medications	460 (69%)	250 (37%)	0.313432836	0.26, 0.37	<0.001	<0.001
Nonsteroidal Anti-inflammatory Agents (NSAIDs)	130 (19%)	486 (73%)	−0.531343284	−0.58, −0.48	<0.001	<0.001
Opioids	398 (59%)	327 (49%)	0.105970149	0.05, 0.16	<0.001	<0.001
Steroids	68 (10%)	219 (33%)	−0.225373134	−0.27, −0.18	<0.001	<0.001

^1^ n (%); ^2^ Two-sample test for equality of proportions; ^3^ CI = Confidence Interval; ^4^ False discovery rate correction for multiple testing.

**Table 3:** Average longevity of use in years of various medication classes in CRPS cases and matched controls.

Characteristic	CRPS, N = 2510 ^1^	Control, N = 2510 ^1^	Difference ^2^	95% CI ^2,3^	*p*-Value ^2^	q-Value ^4^
Medication Class						
Benzodiazepines	0.76 (1.83)	0.37 (1.30)	0.3876494024	0.30, 0.48	<0.001	<0.001
Bisphosphonates	0.11 (0.67)	0.07 (0.50)	0.0342629482	0.00, 0.07	0.041	0.047
Calcitonin	0.00 (0.04)	0.00 (0.03)	0.0007968127	0.00, 0.00	0.5	0.5
Capsaicin	0.08 (0.38)	0.03 (0.22)	0.0537848606	0.04, 0.07	<0.001	<0.001
Neuropathic pain medications	4.06 (5.18)	1.35 (3.17)	2.7147410359	2.5, 3.0	<0.001	<0.001
NSAIDs	3.11 (4.25)	2.21 (3.08)	0.9051792829	0.70, 1.1	<0.001	<0.001
Opioids	3.95 (5.21)	1.30 (2.57)	2.6442231076	2.4, 2.9	<0.001	<0.001
Steroids	1.07 (1.95)	0.66 (1.43)	0.4151394422	0.32, 0.51	<0.001	<0.001
None	0.00 (0.00)	0.00 (0.00)	0.0000000000			

^1^ Mean (SD); ^2^ Welch Two Sample *t*-test; ^3^ CI = Confidence Interval; ^4^ False discovery rate correction for multiple testing.

**Table 4:** Dose comparisons of first and last prescribed opioids in the CRPS group.

Characteristic	First Dose N = 3930	Last Dose N = 3930	Difference ^1^	95% CI ^1,2^	*p*-Value ^1^	q-Value ^3^
Buprenorphine			0.14	−1.5, 1.2	0.8	>0.9
N	60	60				
Average dose in mg (SD)	7 (4)	7 (4)				
codeine			0.00	−8.3, 8.3	>0.9	>0.9
N	8	8				
Mean (SD)	24 (8)	24 (8)				
Fentanyl patch			−5.0	−2.5, 12	0.2	0.8
N	46	46				
Average dose in mcg/hr (SD)	36 (18)	31 (18)				
hydrocodone			0.15	−0.29, −0.01	0.035	0.4
N	828	834				
Average dose in mg (SD)	5 (1)	6 (2)				
hydromorphone			0.03	−0.81, 0.75	>0.9	>0.9
N	68	68				
Average dose in mg (SD)	3 (2)	3 (2)				
meperidine			0.00	−23, 23	>0.9	>0.9
N	11	11				
Average dose in mg (SD)	77 (26)	77 (26)				
methadone			0.29	−0.79, 0.21	0.3	0.8
N	189	189				
Average dose in mg (SD)	7 (2)	7 (3)				
morphine			0.19	−2.4, 2.1	0.9	>0.9
N	199	200				
Average dose in mg (SD)	22 (12)	22 (11)				
oxycodone			0.13	−0.51, 0.25	0.5	>0.9
N	1610	1612				
Average dose in mg (SD)	7 (5)	7 (6)				
oxymorphone			0.00	−9.0, 9.0	>0.9	>0.9
N	14	14				
Average dose in mg (SD)	16 (12)	16 (12)				
tapentadol			9.1	−29, 11	0.4	0.9
N	22	22				
Average dose in mg (SD)	55 (13)	64 (44)				
tramadol			−0.86	−0.08, 1.8	0.074	0.4
N	817	823				
Average dose in mg (SD)	51 (12)	50 (7)				

^1^ Welch two-sample *t*-test; ^2^ CI = Confidence Interval; ^3^ False discovery rate correction for multiple testing.

**Table 5:** Number of subjects in the CRPS and control groups that have been prescribed opioids prior to and after 2017.

	Control						CRPS					
Characteristic	2017 and after N = 427 ^1^	Prior to 2017 N = 905 ^1^	Difference ^2^	95% CI ^2,3^	*p*-Value ^2^	q-Value ^4^	2017 and after, N = 676 ^1^	Prior to 2017, N = 1470 ^1^	Difference ^2^	95% CI ^2,3^	*p*-Value ^2^	q-Value ^4^
Medication												
Opioids	427 (30%)	905 (51%)	−0.2134379233	−0.25, −0.18	<0.001	<0.001	676 (50%)	1470 (79%)	−0.288834326	−0.32, −0.26	<0.001	<0.001

^1^ n (%); ^2^ Two-sample test for equality of proportions; two-sample test for equality of proportions without continuity correction; ^3^ CI = Confidence Interval; ^4^ False discovery rate correction for multiple testing.

## Data Availability

The data presented in this study are available on request from the corresponding author. The data are not publicly available due to it being obtained from the electronic medical record of a single institution.

## Supplementary Materials

The following supporting information can be downloaded at: https://www.mdpi.com/article/10.3390/brainsci13071012/s1, Figure S1: Visual representation of Table 2a. Figure S1: Visual representation of Table 2b; Table S1: Differences between general prescription rates of specific medications amongst CRPS cases and controls. Table S2: CRPS-associated prescriptions of specific medications amongst CRPS cases compared with matched controls.
