# Supplementary material for: Prescription Trends in Complex Regional Pain Syndrome: A Retrospective Case–Control Study
Source: Brain Sci. 2023 Jun 30;13(7):1012. doi: 10.3390/brainsci13071012 (PMC10377480; doi:10.3390/brainsci13071012)

Supplemental Data

Table S1. Differences between general prescription rates of specific medications amongst CRPS cases and controls.

Characteristic	CRPS, N = 2,510[1]	Control, N = 2,510[1]	Difference[2]	95% CI[23]	p-value[2]	q-value[4]
**Medication**						
None	299 (12%)	0 (0%)	0.1191235060	0.11, 0.13	<0.001	<0.001
Alendronate-Cholecalciferol	0 (0%)	1 (<0.1%)	-0.0003984064	0.00, 0.00	>0.9	>0.9
Alendronate Sodium	83 (3.3%)	75 (3.0%)	0.0031872510	-0.01, 0.01	0.6	0.8
Alprazolam	205 (8.2%)	159 (6.3%)	0.0183266932	0.00, 0.03	0.014	0.033
Amitriptyline HCl	633 (25%)	219 (8.7%)	0.1649402390	0.14, 0.19	<0.001	<0.001
Buprenorphine	25 (1.0%)	11 (0.4%)	0.0055776892	0.00, 0.01	0.030	0.067
Buprenorphine HCl	12 (0.5%)	6 (0.2%)	0.0023904382	0.00, 0.01	0.2	0.4
Buprenorphine HCl-Naloxone HCl	39 (1.6%)	31 (1.2%)	0.0031872510	0.00, 0.01	0.4	0.6
Calcitonin (Salmon)	5 (0.2%)	3 (0.1%)	0.0007968127	0.00, 0.00	0.7	>0.9
Capsaicin	154 (6.1%)	52 (2.1%)	0.0406374502	0.03, 0.05	<0.001	<0.001
Celecoxib	229 (9.1%)	101 (4.0%)	0.0509960159	0.04, 0.07	<0.001	<0.001
Chlordiazepoxide	0 (0%)	1 (<0.1%)	-0.0003984064	0.00, 0.00	>0.9	>0.9
Chlordiazepoxide HCl	5 (0.2%)	3 (0.1%)	0.0007968127	0.00, 0.00	0.7	>0.9
Clorazepate Dipotassium	1 (<0.1%)	1 (<0.1%)	0.0000000000	0.00, 0.00	>0.9	>0.9
Codeine Sulfate	8 (0.3%)	2 (<0.1%)	0.0023904382	0.00, 0.01	0.11	0.2
Cyclobenzaprine-Gabapentin	0 (0%)	1 (<0.1%)	-0.0003984064	0.00, 0.00	>0.9	>0.9
Desipramine HCl	13 (0.5%)	4 (0.2%)	0.0035856574	0.00, 0.01	0.052	0.10
Dexamethasone	50 (2.0%)	31 (1.2%)	0.0075697211	0.00, 0.01	0.044	0.090
Dextromethorphan-guaifenesin	189 (7.5%)	137 (5.5%)	0.0207171315	0.01, 0.03	0.003	0.009
Dextromethorphan-quinidine	3 (0.1%)	4 (0.2%)	-0.0003984064	0.00, 0.00	>0.9	>0.9
Dextromethorphan HBr	4 (0.2%)	2 (<0.1%)	0.0007968127	0.00, 0.00	0.7	>0.9
Diazepam	282 (11%)	124 (4.9%)	0.0629482072	0.05, 0.08	<0.001	<0.001
Diclofenac	1 (<0.1%)	3 (0.1%)	-0.0007968127	0.00, 0.00	0.6	0.8
Diclofenac-misoprostol	29 (1.2%)	11 (0.4%)	0.0071713147	0.00, 0.01	0.007	0.017
Diclofenac Potassium	47 (1.9%)	20 (0.8%)	0.0107569721	0.00, 0.02	0.001	0.004
Diclofenac Sodium	546 (22%)	378 (15%)	0.0669322709	0.05, 0.09	<0.001	<0.001
Duloxetine HCl	567 (23%)	204 (8.1%)	0.1446215139	0.12, 0.16	<0.001	<0.001
Etidronate Disodium	1 (<0.1%)	0 (0%)	0.0003984064	0.00, 0.00	>0.9	>0.9
Etodolac	103 (4.1%)	61 (2.4%)	0.0167330677	0.01, 0.03	0.001	0.003
Fenoprofen Calcium	1 (<0.1%)	0 (0%)	0.0003984064	0.00, 0.00	>0.9	>0.9
Fentanyl patch	73 (2.9%)	17 (0.7%)	0.0223107570	0.01, 0.03	<0.001	<0.001
Flurbiprofen	3 (0.1%)	2 (<0.1%)	0.0003984064	0.00, 0.00	>0.9	>0.9
Gabapentin	1,469 (59%)	674 (27%)	0.3167330677	0.29, 0.34	<0.001	<0.001
Gabapentin & Diet Manage Prod	1 (<0.1%)	0 (0%)	0.0003984064	0.00, 0.00	>0.9	>0.9
Gabapentin (Once-Daily)	24 (1.0%)	4 (0.2%)	0.0079681275	0.00, 0.01	<0.001	0.001
Gabapentin Enacarbil	2 (<0.1%)	0 (0%)	0.0007968127	0.00, 0.00	0.5	0.7
Hydrocodone-Acetaminophen	790 (31%)	457 (18%)	0.1326693227	0.11, 0.16	<0.001	<0.001
Hydrocodone-Chlorpheniramine	1 (<0.1%)	0 (0%)	0.0003984064	0.00, 0.00	>0.9	>0.9
Hydrocodone-guaifenesin	2 (<0.1%)	0 (0%)	0.0007968127	0.00, 0.00	0.5	0.7
Hydrocodone-Homatropine	60 (2.4%)	50 (2.0%)	0.0039840637	0.00, 0.01	0.4	0.6

Characteristic	CRPS, N = 2,510[1]	Control, N = 2,510[1]	Difference[2]	95% CI[23]	p-value[2]	q-value[4]
Hydrocodone-Ibuprofen	20 (0.8%)	10 (0.4%)	0.0039840637	0.00, 0.01	0.10	0.2
Hydrocodone Bitartrate	5 (0.2%)	1 (<0.1%)	0.0015936255	0.00, 0.00	0.2	0.4
Hydromorphone HCl	77 (3.1%)	18 (0.7%)	0.0235059761	0.02, 0.03	<0.001	<0.001
Ibandronate Sodium	15 (0.6%)	12 (0.5%)	0.0011952191	0.00, 0.01	0.7	>0.9
Ibuprofen	1,041 (41%)	1,072 (43%)	-0.0123505976	-0.04, 0.02	0.4	0.6
Ibuprofen-Famotidine	3 (0.1%)	2 (<0.1%)	0.0003984064	0.00, 0.00	>0.9	>0.9
Indomethacin	78 (3.1%)	63 (2.5%)	0.0059760956	0.00, 0.02	0.2	0.4
Ketoprofen	4 (0.2%)	1 (<0.1%)	0.0011952191	0.00, 0.00	0.4	0.6
Ketorolac Tromethamine	135 (5.4%)	58 (2.3%)	0.0306772908	0.02, 0.04	<0.001	<0.001
Lorazepam	437 (17%)	201 (8.0%)	0.0940239044	0.08, 0.11	<0.001	<0.001
Meclofenamate Sodium	3 (0.1%)	1 (<0.1%)	0.0007968127	0.00, 0.00	0.6	0.8
Mefenamic Acid	3 (0.1%)	2 (<0.1%)	0.0003984064	0.00, 0.00	>0.9	>0.9
Meloxicam	659 (26%)	473 (19%)	0.0741035857	0.05, 0.10	<0.001	<0.001
Meperidine HCl	12 (0.5%)	1 (<0.1%)	0.0043824701	0.00, 0.01	0.005	0.014
Methadone	6 (0.2%)	0 (0%)	0.0023904382	0.00, 0.00	0.041	0.086
Methadone HCl	195 (7.8%)	22 (0.9%)	0.0689243028	0.06, 0.08	<0.001	<0.001
Methylprednisolone	389 (15%)	256 (10%)	0.0529880478	0.03, 0.07	<0.001	<0.001
Methylprednisolone Acetate	27 (1.1%)	6 (0.2%)	0.0083665339	0.00, 0.01	<0.001	0.002
Methylprednisolone Sodium Succinate	5 (0.2%)	4 (0.2%)	0.0003984064	0.00, 0.00	>0.9	>0.9
Morphine Sulfate	220 (8.8%)	31 (1.2%)	0.0752988048	0.06, 0.09	<0.001	<0.001
Nabumetone	115 (4.6%)	65 (2.6%)	0.0199203187	0.01, 0.03	<0.001	<0.001
Naproxen	767 (31%)	719 (29%)	0.0191235060	-0.01, 0.04	0.15	0.3
Naproxen-Esomeprazole	1 (<0.1%)	1 (<0.1%)	0.0000000000	0.00, 0.00	>0.9	>0.9
Naproxen Sodium	92 (3.7%)	56 (2.2%)	0.0143426295	0.00, 0.02	0.003	0.009
Naproxen Sodium-Gabapentin	1 (<0.1%)	0 (0%)	0.0003984064	0.00, 0.00	>0.9	>0.9
Nortriptyline HCl	206 (8.2%)	67 (2.7%)	0.0553784861	0.04, 0.07	<0.001	<0.001
Oxaprozin	17 (0.7%)	6 (0.2%)	0.0043824701	0.00, 0.01	0.037	0.081
Oxazepam	1 (<0.1%)	0 (0%)	0.0003984064	0.00, 0.00	>0.9	>0.9
Oxycodone	6 (0.2%)	0 (0%)	0.0023904382	0.00, 0.00	0.041	0.086
Oxycodone-Acetaminophen	1,128 (45%)	583 (23%)	0.2171314741	0.19, 0.24	<0.001	<0.001
Oxycodone HCl	553 (22%)	183 (7.3%)	0.1474103586	0.13, 0.17	<0.001	<0.001
Oxymorphone HCl	23 (0.9%)	4 (0.2%)	0.0075697211	0.00, 0.01	<0.001	0.002
Piroxicam	7 (0.3%)	9 (0.4%)	-0.0007968127	0.00, 0.00	0.8	>0.9
Prednisolone	4 (0.2%)	1 (<0.1%)	0.0011952191	0.00, 0.00	0.4	0.6
Prednisolone Acetate	78 (3.1%)	83 (3.3%)	-0.0019920319	-0.01, 0.01	0.7	>0.9
Prednisolone Sodium Phosphate	5 (0.2%)	4 (0.2%)	0.0003984064	0.00, 0.00	>0.9	>0.9
Prednisone	928 (37%)	616 (25%)	0.1243027888	0.10, 0.15	<0.001	<0.001
Pregabalin	738 (29%)	142 (5.7%)	0.2374501992	0.22, 0.26	<0.001	<0.001
Risedronate Sodium	14 (0.6%)	7 (0.3%)	0.0027888446	0.00, 0.01	0.2	0.3
Rofecoxib	0 (0%)	5 (0.2%)	-0.0019920319	0.00, 0.00	0.073	0.14
Sulindac	21 (0.8%)	7 (0.3%)	0.0055776892	0.00, 0.01	0.014	0.033
Tapentadol HCl	42 (1.7%)	3 (0.1%)	0.0155378486	0.01, 0.02	<0.001	<0.001
Tetracycline HCl	1 (<0.1%)	3 (0.1%)	-0.0007968127	0.00, 0.00	0.6	0.8
Topiramate	427 (17%)	163 (6.5%)	0.1051792829	0.09, 0.12	<0.001	<0.001

Characteristic	CRPS, N = 2,510[1]	Control, N = 2,510[1]	Difference[2]	95% CI[23]	p-value[2]	q-value[4]
Tramadol-Acetaminophen	20 (0.8%)	5 (0.2%)	0.0059760956	0.00, 0.01	0.005	0.013
Tramadol HCl	822 (33%)	394 (16%)	0.1705179283	0.15, 0.19	<0.001	<0.001
Valdecoxib	0 (0%)	2 (<0.1%)	-0.0007968127	0.00, 0.00	0.5	0.7
Zoledronic Acid	2 (<0.1%)	5 (0.2%)	-0.0011952191	0.00, 0.00	0.4	0.7

[1]n (%); [2]Two sample test for equality of proportions; 2-sample test for equality of proportions without continuity correction; [3]CI = Confidence Interval; [4]False discovery rate correction for multiple testing.

Table S2. CRPS-associated prescriptions of specific medications amongst CRPS cases compared with matched controls.

Characteristic	CRPS, N = 670[1]	Other, N = 670[1]	Difference[2]	95% CI[2,3]	p-value[2]	q-value[4]
**Medication**						
Alendronate Sodium	1 (0.1%)	19 (2.8%)	-0.026865672	-0.04, -0.01	<0.001	<0.001
Alprazolam	11 (1.6%)	33 (4.9%)	-0.032835821	-0.05, -0.01	0.001	0.005
Amitriptyline HCl	85 (13%)	53 (7.9%)	0.047761194	0.01, 0.08	0.005	0.019
Buprenorphine	8 (1.2%)	5 (0.7%)	0.004477612	-0.01, 0.02	0.6	0.9
Buprenorphine HCl	3 (0.4%)	1 (0.1%)	0.002985075	0.00, 0.01	0.6	0.9
Buprenorphine HCl-Naloxone HCl	1 (0.1%)	7 (1.0%)	-0.008955224	-0.02, 0.00	0.076	0.2
Calcitonin	1 (0.1%)	0 (0%)	0.001492537	0.00, 0.01	>0.9	>0.9
Capsaicin	7 (1.0%)	19 (2.8%)	-0.017910448	-0.03, 0.00	0.029	0.084
Celecoxib	19 (2.8%)	24 (3.6%)	-0.007462687	-0.03, 0.01	0.5	0.8
Chlordiazepoxide HCl	0 (0%)	2 (0.3%)	-0.002985075	-0.01, 0.00	0.5	0.8
Desipramine HCl	2 (0.3%)	0 (0%)	0.002985075	0.00, 0.01	0.5	0.8
Dexamethasone	0 (0%)	9 (1.3%)	-0.013432836	-0.02, 0.00	0.007	0.026
Dextromethorphan-guaifenesin	0 (0%)	45 (6.7%)	-0.067164179	-0.09, -0.05	<0.001	<0.001
Dextromethorphan-quinidine	0 (0%)	1 (0.1%)	-0.001492537	-0.01, 0.00	>0.9	>0.9
Diazepam	18 (2.7%)	35 (5.2%)	-0.025373134	-0.05, 0.00	0.025	0.078
Diclofenac	0 (0%)	2 (0.3%)	-0.002985075	-0.01, 0.00	0.5	0.8
Diclofenac-misoprostol	2 (0.3%)	6 (0.9%)	-0.005970149	-0.02, 0.00	0.3	0.6
Diclofenac Potassium	5 (0.7%)	10 (1.5%)	-0.007462687	-0.02, 0.01	0.3	0.6
Diclofenac Sodium	15 (2.2%)	104 (16%)	-0.132835821	-0.16, -0.10	<0.001	<0.001
Duloxetine HCl	58 (8.7%)	54 (8.1%)	0.005970149	-0.03, 0.04	0.8	>0.9
Etodolac	6 (0.9%)	20 (3.0%)	-0.020895522	-0.04, 0.00	0.010	0.033
Fentanyl patch	9 (1.3%)	1 (0.1%)	0.011940299	0.00, 0.02	0.026	0.079
Flurbiprofen	1 (0.1%)	0 (0%)	0.001492537	0.00, 0.01	>0.9	>0.9
Gabapentin	237 (35%)	181 (27%)	0.083582090	0.03, 0.13	0.001	0.005
Gabapentin (Once-Daily)	2 (0.3%)	1 (0.1%)	0.001492537	-0.01, 0.01	>0.9	>0.9
Hydrocodone-Acetaminophen	69 (10%)	121 (18%)	-0.077611940	-0.12, -0.04	<0.001	<0.001
HYDROcodone-Homatropine	0 (0%)	11 (1.6%)	-0.016417910	-0.03, -0.01	0.002	0.009
Hydrocodone -Ibuprofen	2 (0.3%)	2 (0.3%)	0.000000000	-0.01, 0.01	>0.9	>0.9
Hydrocodone Bitartrate	1 (0.1%)	0 (0%)	0.001492537	0.00, 0.01	>0.9	>0.9
Hydromorphone HCl	12 (1.8%)	8 (1.2%)	0.005970149	-0.01, 0.02	0.5	0.8
Ibandronate Sodium	0 (0%)	2 (0.3%)	-0.002985075	-0.01, 0.00	0.5	0.8

Characteristic	CRPS, N = 670[1]	Other, N = 670[1]	Difference[2]	95% CI[2,3]	p-value[2]	q-value[4]
Ibuprofen	32 (4.8%)	294 (44%)	-0.391044776	-0.43, -0.35	<0.001	<0.001
Ibuprofen-Famotidine	0 (0%)	1 (0.1%)	-0.001492537	-0.01, 0.00	>0.9	>0.9
Indomethacin	1 (0.1%)	17 (2.5%)	-0.023880597	-0.04, -0.01	<0.001	0.002
Ketorolac Tromethamine	2 (0.3%)	9 (1.3%)	-0.010447761	-0.02, 0.00	0.069	0.2
Lorazepam	76 (11%)	57 (8.5%)	0.028358209	-0.01, 0.06	0.10	0.2
Mefenamic Acid	0 (0%)	1 (0.1%)	-0.001492537	-0.01, 0.00	>0.9	>0.9
Meloxicam	34 (5.1%)	134 (20%)	-0.149253731	-0.19, -0.11	<0.001	<0.001
Meperidine HCl	2 (0.3%)	0 (0%)	0.002985075	0.00, 0.01	0.5	0.8
Methadone HCl	63 (9.4%)	3 (0.4%)	0.089552239	0.07, 0.11	<0.001	<0.001
Methylprednisolone	11 (1.6%)	62 (9.3%)	-0.076119403	-0.10, -0.05	<0.001	<0.001
Methylprednisolone Acetate	12 (1.8%)	3 (0.4%)	0.013432836	0.00, 0.03	0.038	0.10
Methylprednisolone Sodium Succ	0 (0%)	1 (0.1%)	-0.001492537	-0.01, 0.00	>0.9	>0.9
Morphine Sulfate	51 (7.6%)	9 (1.3%)	0.062686567	0.04, 0.09	<0.001	<0.001
Nabumetone	7 (1.0%)	16 (2.4%)	-0.013432836	-0.03, 0.00	0.092	0.2
Naproxen	23 (3.4%)	188 (28%)	-0.246268657	-0.28, -0.21	<0.001	<0.001
Naproxen Sodium	0 (0%)	19 (2.8%)	-0.028358209	-0.04, -0.01	<0.001	<0.001
Nortriptyline HCl	17 (2.5%)	20 (3.0%)	-0.004477612	-0.02, 0.01	0.7	>0.9
Oxaprozin	1 (0.1%)	1 (0.1%)	0.000000000	0.00, 0.00	>0.9	>0.9
Oxycodone	2 (0.3%)	0 (0%)	0.002985075	0.00, 0.01	0.5	0.8
Oxycodone-Acetaminophen	170 (25%)	169 (25%)	0.001492537	-0.05, 0.05	>0.9	>0.9
Oxycodone HCl	82 (12%)	60 (9.0%)	0.032835821	0.00, 0.07	0.062	0.2
Oxymorphone HCl	3 (0.4%)	1 (0.1%)	0.002985075	0.00, 0.01	0.6	0.9
Piroxicam	0 (0%)	2 (0.3%)	-0.002985075	-0.01, 0.00	0.5	0.8
Prednisolone	1 (0.1%)	0 (0%)	0.001492537	0.00, 0.01	>0.9	>0.9
Prednisolone Acetate	0 (0%)	22 (3.3%)	-0.032835821	-0.05, -0.02	<0.001	<0.001
Prednisolone Sodium Phosphate	0 (0%)	1 (0.1%)	-0.001492537	-0.01, 0.00	>0.9	>0.9
Prednisone	46 (6.9%)	167 (25%)	-0.180597015	-0.22, -0.14	<0.001	<0.001
Pregabalin	182 (27%)	39 (5.8%)	0.213432836	0.17, 0.25	<0.001	<0.001
Risedronate Sodium	0 (0%)	2 (0.3%)	-0.002985075	-0.01, 0.00	0.5	0.8
Rofecoxib	0 (0%)	1 (0.1%)	-0.001492537	-0.01, 0.00	>0.9	>0.9
Sulindac	3 (0.4%)	2 (0.3%)	0.001492537	-0.01, 0.01	>0.9	>0.9
Tapentadol HCl	8 (1.2%)	1 (0.1%)	0.010447761	0.00, 0.02	0.045	0.12
Tetracycline HCl	0 (0%)	1 (0.1%)	-0.001492537	-0.01, 0.00	>0.9	>0.9
Topiramate	46 (6.9%)	50 (7.5%)	-0.005970149	-0.04, 0.02	0.8	>0.9
Tramadol-Acetaminophen	1 (0.1%)	2 (0.3%)	-0.001492537	-0.01, 0.01	>0.9	>0.9
Tramadol HCl	100 (15%)	103 (15%)	-0.004477612	-0.04, 0.04	0.9	>0.9
Valdecoxib	0 (0%)	2 (0.3%)	-0.002985075	-0.01, 0.00	0.5	0.8
Zoledronic Acid	0 (0%)	2 (0.3%)	-0.002985075	-0.01, 0.00	0.5	0.8

[1]n (%); [2]Two sample test for equality of proportions; 2-sample test for equality of proportions without continuity correction; [3]CI = Confidence Interval; [4]False discovery rate correction for multiple testing.

Figure S1: Visual representation of Table 2a

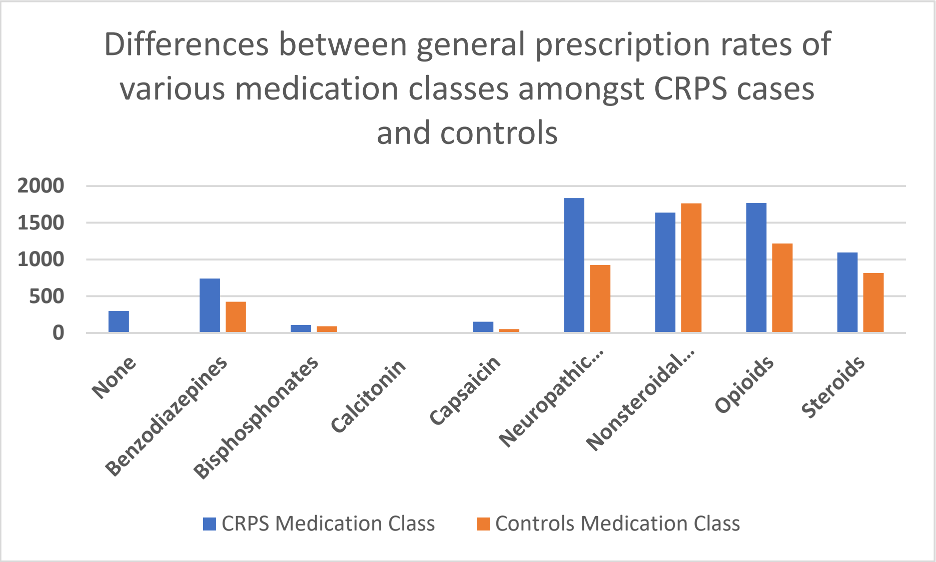

Figure S2. Visual representation of Table 2b.

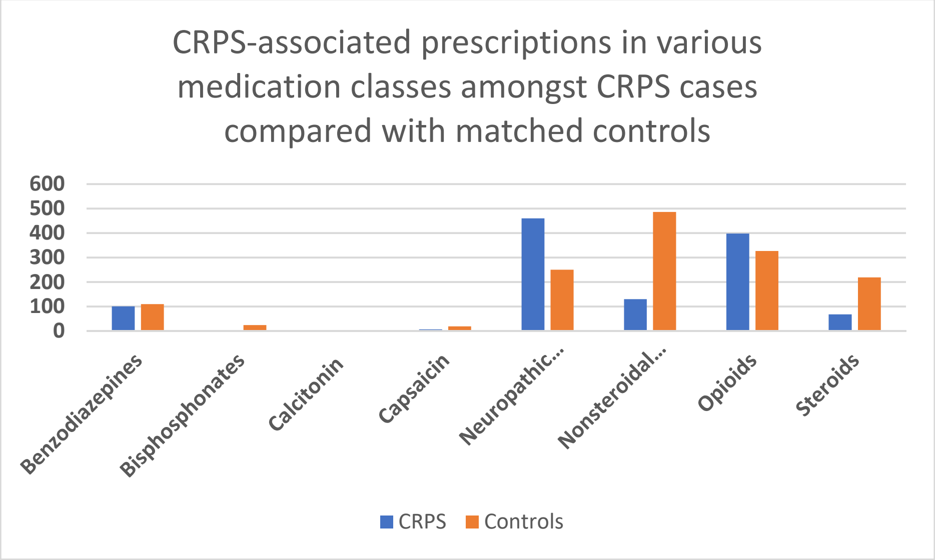

Supplement: Supplementary file 1 [file brainsci-13-01012-s001.zip › brainsci-2464441-supplementary.pdf]
